# Chimeric Antigen Receptor T-Cell Therapy for Multiple Myeloma

**DOI:** 10.3390/cancers11122024

**Published:** 2019-12-15

**Authors:** Naoki Hosen

**Affiliations:** Department of Cancer Stem Cell Biology, Osaka University Graduate School of Medicine, Suita, 1-7 Yamada-Oka, Suita, Osaka 565-0871, Japan; hnaoki@imed3.med.osaka-u.ac.jp; Tel.: +81-06-6879-3676; Fax: +81-06-6879-3677

**Keywords:** CAR T cell, immunotherapy, multiple myeloma, integrin

## Abstract

CD19 Chimeric antigen receptor (CAR) T cell therapy has been shown to be effective for B cell leukemia and lymphoma. Many researchers are now trying to develop CAR T cells for various types of cancer. For multiple myeloma (MM), B-cell maturation antigen (BCMA) has been recently proved to be a promising target. However, cure of MM is still difficult, and several other targets, for example immunoglobulin kappa chain, SLAM Family Member 7 (SLAMF7), or G-protein coupled receptor family C group 5 member D (GPRC5D), are being tested as targets for CAR T cells. We also reported that the activated integrin β7 can serve as a specific target for CAR T cells against MM, and are preparing a clinical trial. In this review, we summarized current status of CAR T cell therapy for MM and discussed about the future perspectives.

## 1. Chimeric antigen Receptor (CAR) T-Cell Therapy

**Figure 1:**
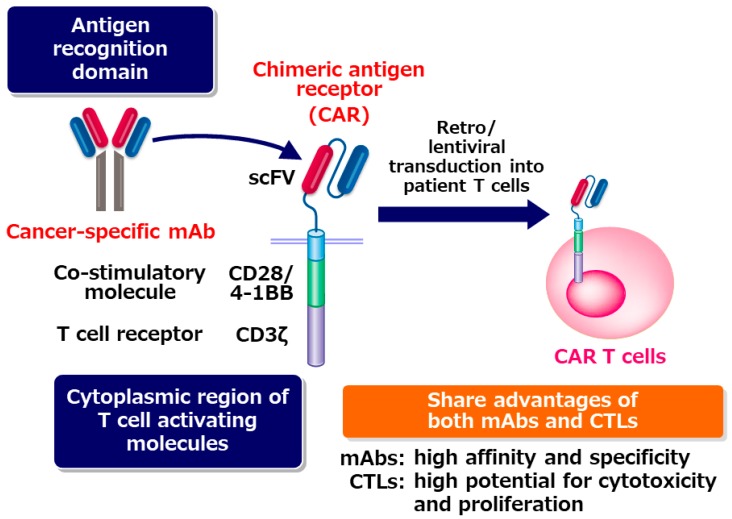
CAR T cells share the advantages of both monoclonal antibodies (mAbs) and cytotoxic T cells. CTL: Cytotoxic T cell.

The big success of checkpoint blockade therapy revealed that autologous T cells in cancer patients have extremely high potential to eradicate tumor cells once they can recognize tumor cell as targets. In CAR T cells, the antigen recognition domain of a tumor-specific monoclonal antibody (mAb) is used for letting T cells recognize tumor cells. Antigen-recognition domain of the mAb is fused with co-stimulatory molecule such as CD28 or 4-1BB and CD3ζ to generate CAR. CAR T cells are established by transducing the CAR cDNA into a patient’s T cells. CAR-transduced T cells are expanded in vitro, and then infused into the patient. CAR T cells can target tumor cells specifically, similar to mAb drugs. Different from mAb drugs, CAR T cells can expand extensively when they are activated upon recognition of the tumor cells [1] (Figure 1).

CD19 CAR T cell therapy has been proven to be effective for acute lymphoblastic leukemia and B cell lymphoma [2,3,4]. Initially, CAR T cell therapy was thought to be dangerous because it frequently induced severe cytokine syndrome (CRS) and was sometimes fatal [5]. However, tocilizumab (anti-IL6 receptor mAb) was found to be highly effective for CRS. CRS can be controlled by appropriate usage of tocilizumab. Importantly, the major source of IL-6 is activated macrophages but not T cells, suggesting that cytotoxicity of CAR T cells is not impaired by blocking IL6 signal [6,7].

## 2. BCMA CAR T-Cell Therapy for Multiple Myeloma

Multiple myeloma (MM) is one of the most frequent hematological cancers, and is characterized by aberrant expansion of clonal plasma cells. Proteasome inhibitors and immunomodulatory drugs such as lenalidomide largely improves the prognosis of MM patients [8]. In addition, antibody drugs against CD38 and CS1 showed remarkable effect [9,10,11]. However, the cure of MM is still extremely difficult, and relapsed and refractory MM patients have poor prognosis. Therefore, development of new therapeutic drugs is urgently needed. CAR T-cell therapy is considered one of the most promising strategies for curing MM.

B-cell maturation antigen (BCMA) has been recently proved to be a promising antigen for CAR T cells against MM. BCMA is specifically expressed in MM cells in most MM patients. BCMA is not expressed in hematopoietic stem and progenitor cells, and non-hematopoietic vital organs. CAR T-cell therapy targeting BCMA has been already tested in clinical trials (Table 1).

Carpenter et al. developed an anti-BCMA CAR using CD28 as a co-stimulatory molecule [18] and performed a phase I dose-escalation study [12,19]. Relapsed/refractory MM patients received preconditioning regimen with cyclophosphamide and fludarabine, and then, they were infused with BCMA CAR T cells. Sixteen patients received the highest dose of 9 × 10^6^ CAR-BCMA T cells/kg. The overall response rate was 81%. Very good partial response (VGPR) or complete response was observed in 63% of the patients. The median event-free survival was 31 weeks. In bone marrow of all 11 patients who had partial response or better, minimal residual disease (MRD) was not detected. In some patients, especially those that received the highest does of BCMA CAR T cells, severe CRS occurred in some cases, but all the patients recovered from CRS.

BCMA-targeting CAR T cells were also generated in the University of Pennsylvania using 4-1BB as a co-stimulatory molecule [13]. Lentivirus was used for transduction of the CAR into T cells. BCMA-specific CAR was fully humanized [13]. In the clinical trial, 25 patients comprised three cohorts depending on their treatment, as follows: (1) 1–5 × 10^8^ CART-BCMA cells alone; (2) cyclophosphamide (Cy) 1.5 g/m^2^ + 1–5 × 10^7^ CART-BCMA cells; and (3) Cy 1.5 g/m^2^ + 1–5 × 10^8^ CART-BCMA cells. The numbers of treated subjects exhibiting responses were 4/9 (44%) in cohort 1, 1/5 (20%) in cohort 2, and 7/11 (64%) in cohort 3; these included five partial, five very good partial, and two complete responses. CRS was observed in 88% of subjects and was ≥ grade 3 in 32%. Three patients (12%) had ≥ grade 3 neurotoxicity. One patient died during the trial after developing grade 4 CRS complicated by candidemia.

Promising results of a multicenter phase I study of bb2121 were recently reported [14]. Bb2121 consists of CAR T cells transduced with a lentiviral vector encoding an anti-BCMA single-chain variable fragment (scFv). In Bb2121, 4-1BB was used for costimulatory molecules. Patients with relapsed and refractory MM who had received ≥ 3 prior lines of therapy, including a proteasome inhibitor and an immunomodulatory drug, were enrolled. Pre-conditioning with fludarabine and Cy was performed before injection of bb2121. Patients were administered bb2121 as a single infusion at doses of 50 × 10^6^, 150 × 10^6^, 450 × 10^6^, or 800 × 10^6^ CAR-positive T cells in the dose-escalation phase, and 150 × 10^6^ to 450 × 10^6^ CAR+ T cells in the expansion phase. The response rate was 85%, including 15 patients (45%) with complete responses. Relapse was observed in six out of the 15 patients who had a complete response. The median progression-free survival (PFS) was 11.8 months. MRD (≤10^−4^ nucleated cells) was not detected in all 16 responders (partial response or better). In two patients, severe CRS (grade 3) occurred (6%), but all patients recovered from CRS. There were no grade 4 or 5 CRS events. Grade 4 neurotoxicity was observed in one patient (3%).

LCAR-B38M, a dual-epitope binding CAR T-cell therapy was developed by Nanjing Legend Biotech in China. Recently, the results of the first clinical trial were reported [15]. This CAR recognizes two independent epitopes in BCMA. LCAR-B38M were infused to 17 patients with relapsed/refractory MM. Stringent complete response (sCR) was achieved in 13 patients, and very good partial response (VGPR) was achieved in two patients. Eight patients remained sCR with a median follow-up of 417 days. CRS occurred in six patients but were manageable, while one patient died of severe toxic reaction.

In Memorial Sloan Kettering, MCARH171, which is composed of a CD3ζ and 4-1BB stimulatory domains, and an anti-BCMA scFV, was tested. In two of five patients who received the high dose (>450 × 106 cells) of MCARH171, continuous very good PR was observed [16].

In Tongji Hospital of Tongji Medical College, BRD015, a BCMA-targeted CAR-T product containing a murine anti-BCMA scFv and CD28 were tested. In the patients with high BCMA expression, the ORRs were 87% (73% CR) [17].

All of these trials clearly demonstrate that CAR T cells targeting BCMA is effective against relapsed/refractory MM. The frequencies of severe CRS and neurotoxicity are comparable to those observed in CD19 CAR T-cell therapy. These results suggest that BCMA CAR T-cell therapy is promising for relapsed/refractory MM. However, thus far, the long term efficacy of BCMA CAR T-cell is unsatisfactory, and curing MM is still difficult. Therefore, several researchers including us are now searching for additional targets.

The mechanisms for relapse after BCMA CAR T-cell therapy is now being analyzed extensively. One major mechanism is obviously the escape of BCMA^low^ MM cells. To enhance the expression of BCMA on the cell surface, gamma secretase inhibitor may be useful for inhibiting shedding of BCMA from cell surface [20]. Another reason for the relapse is short persistence of BCMA CAR T-cells in vivo. Many researchers are now trying to develop CAR T-cells that has the potential to persist for longer period in vivo. Recent findings about the mechanisms for T cell exhaustion [21,22,23,24] may be helpful for developing such CAR T-cells.

## 3. CD19-Positive B Cells as Candidates for Therapeutic Targets to Cure MM

The origin of MM is still controversial. Variable regions of MM plasma cells have somatic hyper-mutations, but do not have varieties in the mutation in a patient, suggesting that MM clones are derived from post-germinal center B cells [25]. It has been also shown that clonotypic B cells that share immunoglobulin sequences with MM cells can be detected in MM patients, and are sometimes expanded extensively [26,27]. MM disease could be reconstituted in immune-deficient mice transplanted with CD19^+^ B cells from some MM patients, suggesting that MM stem cells may exist in the CD19^+^ B cell population [28]. However, following reports showed that reconstitution of MM disease were rarely observed upon transplantation of the CD19^+^ B cells from MM patients into the mice, while reconstitution of MM disease by transplantation with CD19^-^CD38^high^ MM plasma cells from many patients was observed in SCID-hu or SCID-rab model [29,30]. Although thus far, we do not have evidence showing that CD19^+^ clonotypic B cells are MM stem or progenitor cells in most MM patients, it should be noted that the lack of engraftment of CD19^+^ clonotypic B cells in immune-deficient mice does not necessarily mean that those cells are not MM stem or precursor cells. It is certainly true that clonotypic B cells sharing immunoglobulin sequences with MM cells exist in MM patients, and those cells are still candidates for MM precursor cells. Clinical efficacy of CAR T cells that can target CD19^+^ clonotypic B cells in MM patients may tell us the significance of CD19^+^ clonotypic B cells for maintaining MM clones.

## 4. Clinical Trial of CAR T-Cell Therapy Targeting CD19

CD19 CAR T cell therapy as a salvage therapy after autologous stem cell transplantation (ASCT) for patients with relapsed/refractory MM patients was reported by Garfall et al. [31,32]. Patients who had relapsed/refractory MM, had previously undergone ASCT, and had a PFS of less than one year were enrolled in the trial. After high-dose melphalan and ASCT, patients received CD19 CAR T cells. In the first report, continuous complete remission was observed in a patient who received the second ASCT plus CD19 CAR T cell therapy, although the first ASCT was not effective [32]. In the following report, significantly longer progression free survival after ASCT + CTL019 compared with prior ASCT was observed in two out of 10 patients (479 versus 181 days and 249 versus 127 days, respectively). These results suggest that CAR T therapy targeting CD19^+^ B cells following ASCT may be beneficial in some patients, although trials in larger numbers of patients will be needed to confirm the results.

Cocktail infusion of CAR T cells targeting different antigens may be useful to avoid immune escape by antigen loss. In addition, in the case of MM depleting CD19^+^ clonotypic B cells may be useful to inhibit relapse as discussed in the previous section. Combinatorial CAR T cell therapy with CD19 CAR T cell and BCMA CAR T cells was also tested by Yan et al. [33]. Patients with relapsed or refractory MM were administered with fludarabine and cyclophosphamide before infusion of CD19 CAR T cells and BCMA CAR T cells. Among 21 patients, nine patients (43%) had stringent complete responses, three (14%) had complete responses, and five (24%) had very good partial responses. CRS occurred in 19 out of 21 patients, but were mild in most cases. One patient died due to cerebral hemorrhage, which was considered related to sustained thrombocytopenia. These results suggest that combinatorial therapy with CD19 CAR T and BCMA CAR T cells is promising, and should be tested with more patients.

## 5. Development of CAR T-Cells Targeting Antigens other than BCMA and CD19

CAR T cells targeting several targets other than BCMA or CD19 are being developed (Table 2). CAR T cells targeting immunoglobulin kappa light chain were tested for non-Hodgkin lymphoma/chronic lymphocytic leukemia or MM patients. Stable disease was observed in five out of eight MM patients [34]. In addition, SLAMF7 is also shown to be a good target for CAR T cells against MM cells [35]. CAR T cells that are generated by fusing APRIL, a ligand for BCMA and TACI, with T cell-activating molecules are also shown to be effective in pre-clinical experiments [36]. Smith et al. in Memorial Sloan Kettering Center recently reported GPRC5 as a promising target for CAR T cells against MM [37].

**Figure 2:**
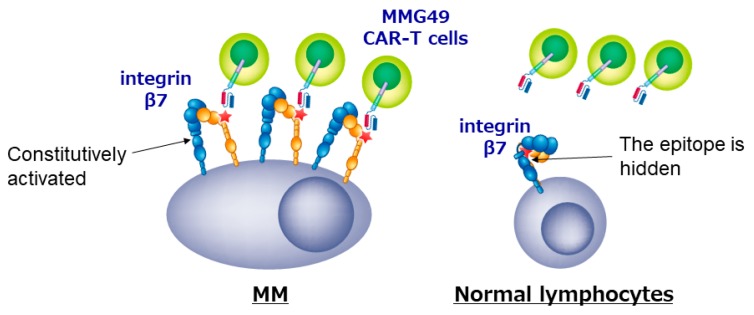
CAR T cells targeting the activated conformation of integrin β7 is promising for MM.

We also developed a new CAR T cell for MM. Since genes and proteins that are specifically expressed in MM cells have been thoroughly searched for using transcriptome or proteome analyses, no MM-specific transcripts or proteins seems to remain unidentified anymore. However, we cannot find differences in post-translational change such as glycosylation or conformational changes using the comprehensive analyses, and may have missed MM-specific antigens formed as a result of post-translational events. For example, Posey et al. recently reported that a cancer-specific glyco-epitope on the Muc1 protein (Tn-Muc1) can serve as a target for CAR T cells against several cancers [39]. To identify MM-specific antigen formed by post-translational events, we first tried to isolate MM-specific mAbs as many as possible, and then characterized the antigens recognized by those MM-specific mAbs. We established more than 10,000 clones of mAbs that reacted with MM cell lines, selected ones that bound to MM cells but not to normal peripheral blood mononuclear cells, and got approximately 500 MM-specific mAbs. Then, we stained BM cells from MM patients with those candidate mAbs, and finally, found that a mAb MMG49 specifically bound to MM cells but not to CD45^+^ normal leukocytes in BM of most MM patients. We also found that MMG49 react with integrin β7 protein by expression cloning. Interestingly, although integrin β7 is expressed in normal lymphocytes, MMG49 does not react with them. We clarified the mechanism for the MM-specificity of MMG49. MMG49 recognizes an epitope exposed only when integrin β7 adopts the activated conformation. We also found that integrin β7 constitutively adopts the activated conformation in MM cells. Therefore, MMG49 abundantly binds to MM cells. Although integrin β7 is also expressed in normal lymphocytes at low levels, it adopts the inactive conformation in most situations to avoid unnecessary adhesion. Thus, MMG49 rarely binds to normal lymphocytes. In addition, integrin β7 mRNA is not detected in non-hematopoietic tissues. These results indicate that MMG49 is a mAb that is highly specific for MM cells. The MMG49 antigen is expressed in not only mature MM cells but also CD19^+^ B cells in BM of MM patients. We proved that the sequences of immunoglobulin expressed in MM cells were also detected in the MMG49^+^ CD19^+^ B cells from the same patient, suggesting that clonotypic CD19^+^ B cells, which are the candidates for MM precursor cells, express the MMG49 antigens. Taking these advantages, we generated CAR T cells using the antigen recognition domain of MMG49. The resultant MMG49 CAR T cells eradicated MM cells when administered into mice engrafted with MM cells. MMG49 did not damage normal human hematopoietic cells engrafted in immune-deficient mice. These results suggested that the MMG49 CAR T cell therapy is promising for MM (Figure 2), and now, a clinical trial is being prepared. More importantly, these results provide the first clear evidence that a receptor protein with a rare but physiologically relevant conformation can serve as a target for cancer immunotherapy [38].

## 6. Future Perspective

### 6.1. Improvement of Trafficking of CAR T Cells to Tumor Sites

MM cells sometimes forms extra-medullary tumors. To eliminate extra-medullary tumors, CAR T cells have to traffic to tumor sites efficiently. However, CAR T cell therapy has thus far not been effective for solid tumors, and inefficient trafficking of CAR T cells to tumor sites is one major reason for the ineffectiveness. Several efforts are being put into improving CAR T cell trafficking to tumor sites. Among them, one promising approach is “prime CAR T cell”, reported by Adachi et al. [40]. They engineered CAR-T cells to express interleukin (IL)-7 and CCL19, which are essential for maintaining T-cell zones in lymph nodes, and named them as 7 × 19 CAR-T cells. The anti-tumor effect of 7 × 19 CAR-T cells were significantly higher than that of conventional CAR-T cells. Interestingly and importantly, 7 × 19 CAR T cells not only directly eliminate tumor cells but also induce response of endogenous T cells to tumor cells. Destruction of tumors by CAR T cells is likely to induce tumor antigen spreading and elicit T cell response to many tumor antigens.

### 6.2. Enhancement of Persistence of CAR T Cells In Vivo

Persistence of CAR T cells in vivo is an important factor for avoiding relapse after CAR T cell therapy. Therefore, many researchers are now trying to develop CAR construct that can induce long persistence of CAR T cell in vivo. One promising approach has been recently reported by Kagoya et al. [41]. Multiple signals, including T cell receptor (TCR) engagement (signal 1), co-stimulation (signal 2), and cytokine engagement (signal 3) are required for optimal TCR signaling. However, current CAR constructs does not contain a domain that transduces cytokine signals. To transduce cytokine signal, they put a truncated cytoplasmic domain of IL2 receptor beta-chain and a STAT3-binding in the CAR construct and named it as the 28-DeltaIL2RB-z(YXXQ) CAR. The 28-DeltaIL2RB-z(YXXQ) CAR activate the JAK/STAT pathway upon co-culturing with tumor antigen-positive cells. Importantly, compared with the conventional CAR T cells, the 28-DeltaIL2RB-z(YXXQ) CAR T cells showed less differentiated phenotype. Furthermore, the 28-DeltaIL2RB-z(YXXQ) CAR-T cells persists for longer than conventional CAR T cells in vivo. Reflecting the improved CAR T cell function, the anti-tumor effect of the 28-DeltaIL2RB-z(YXXQ) CAR-T cells is significantly better than that of conventional CAR T cells.

### 6.3. Development of CAR T Cell Therapy without CRS

An ideal CAR T cells are ones that have high anti-tumor effect but do not induce severe cytokine syndrome. Recently, Ying et al. reported safe and potent CD19 CAR T cells [42]. They tested the potential of several CD19 CAR constructs that have different length of the extracellular and intracellular domains of the CD8α molecule. They selected the CD19-BBz (86) variant CAR, which contains longer extracellular and intracellular sequences from CD8α than the conventional CD19 CAR, as ones that produced low levels of cytokines and expressed high levels of anti-apoptotic molecules. In a clinical trial of CD19-BBz (86) CAR T cell therapy in patients with B cell lymphoma, complete response was achieved in six out of 11 patients (54.5%). Surprisingly, serum cytokine levels were not elevated after CAR T cell infusion in all patients, and consequently, no CRS occurred. These results suggest the possibility of developing CAR T cells without severe side effect.

### 6.4. Development of off the Shelf CAR T Cells

Because thus far CAR T cells have had to be made in an autologous setting, the cost for this therapy is extremely high. “Off the shelf” CAR T cells, which are established from a donor and can be used for many patients, may resolve this issue. To use an allogenic donor is one strategy [43]. Universal CAR19 (UCART19) T cells were generated by deleting endogenous T cell receptors using genome editing mediated by transcription activator-like effector nuclease (TALEN). Two infants with B cell leukemia received UCART19 after lympho-depletion chemotherapy. Both patients achieved CR and then received an allogenic hematopoietic stem cell transplant. This bridge-to-transplantation strategy demonstrates the therapeutic potential of gene-editing technology. To use induced pluripotent stem (iPS )cells for the source of the T cells is another strategy. It has been already reported that functional T cells can be generated from iPS cells [44,45]. Now, many researchers have putting in effort to use them for the source of CAR T cells.

### 6.5. Armored CAR T Cells

One strategy for improving the effectiveness of CAR T cells is to allow CAR T cells to express molecules that make tumor immunity easier to work. For example, the effectiveness of CAR T cells expressing IL-12 has been reported [46]. IL-12 is one of the most powerful anti-cancer cytokines and can act through pleiotropic effects on both innate and acquired immune cells to remodel the tumor micro-environment. A similar idea has been reported for CAR T cells that secrete IL-18 and HVEM [47], or CD40 ligand [48]. Recently, there has also been a report from CAR that emphasizes the function of a drug “carrier” to improve the microenvironment rather than the cytotoxicity of CAR T cells. For example, a system that produces checkpoint antibodies or bispecific antibodies in response to a stimulus from CAR, or a molecule that stimulates the innate immune system, such as flagellin [49]. By using this system, these tumor immunity promoting factors can be expressed only at the tumor site, so that the influence on the whole body can be minimized.

### 6.6. Avoidance of T Cell Exhaustion

Under the conditions of chronic antigen exposure and inflammation, T cells are unable to functionally express effector activity, resulting in a condition called T cell exhaustion [50]. In many cancer patients, especially in the tumor microenvironment, tumor-specific T cells are exhausted [51]. Recently, the molecular mechanisms for T cell exhaustion has been clarified. Tox [21,24] and NR4a [22] were reported to be critical regulators of T cell exhaustion. Thus, we may be able to avoid CAR T cell exhaustion by deleting those factors using genome editing techniques.

### 6.7. T Lymphocyte Metabolism

In recent years, it has been revealed that metabolism is important for T cell differentiation and functional expression. In the tumor microenvironment, tumor cells compete against T cells for the usage of glucose, thereby suppressing T cell function [52]. It was also reported that high levels of potassium released from dead tumor cells strongly inhibits T cell function [53]. Thus, the decline in availability of specific nutrients and the accumulation of metabolic wastes cooperate to change the microenvironment and adversely affect T cell function. Now, many researches are searching for the method to enhance the efficacy of CAR T cell therapy or other T cell-mediated immunotherapy by modulating of metabolism of T cells.

## Figures and Tables

**Table 1:** B cell maturation antigen (BCMA) CAR T-cell therapy trials.

Trial	Construct	ORR (Optimal Doses)	VGPR/CR (Optimal Doses)	References
NCI	Murine, CD28	81% (13/16)	63% (10/16)	[12]
UPENN	Human, 4-1BB	64% (7/11)	36% (4/11)	[13]
Bluebird	Human, 4-1BB	96% (21/22)	86% (19/22)	[14]
Nanjing Legend Biotech	Murine, 4-1BB	88.2% (15/17)	88.2% (15/17)	[15]
Memorial Sloan Kettering	Human 4-1BB	64%	2/5 ongoing VGPR (7.5, 10 mo) (high does cohort (>450 × 10^6^ cells)	[16]
Tongji Hospital of Tongji Medical College	Murine CD28	87%	73% CR	[17]

**Table 2:** Anti-MM CAR T cell targeting antigens other than BCMA and CD19.

Target	Construct	Clinical trial	Response	References
Kappa light chain	CD28	Done	5/8: SD	[34]
SLAMF7	CD28	Not reported yet	-	[35]
BCMA/TACI (APRIL)	CD28	Not reported yet	-	[36]
GPRC5D	4-1BB	Not reported yet	-	[37]
Activated integrin β7	CD28	Not reported yet	-	[38]
